# Effects of Climate Change on Avalanche Accidents and Survival

**DOI:** 10.3389/fphys.2021.639433

**Published:** 2021-04-12

**Authors:** Giacomo Strapazzon, Jürg Schweizer, Igor Chiambretti, Monika Brodmann Maeder, Hermann Brugger, Ken Zafren

**Affiliations:** ^1^Institute of Mountain Emergency Medicine, Eurac Research, Bolzano, Italy; ^2^International Commission for Mountain Emergency Medicine (ICAR MedCom), Zürich, Switzerland; ^3^WSL Institute for Snow and Avalanche Research SLF, Davos, Switzerland; ^4^AINEVA Interregional Association for Coordination and Documentation of Snow and Avalanche Problems, Trento, Italy; ^5^Department of Emergency Medicine, Inselspital University Hospital Bern and Bern University, Bern, Switzerland; ^6^Department of Emergency Medicine, Alaska Native Medical Center, Anchorage, AK, United States; ^7^Department of Emergency Medicine, Stanford University Medical Center, Stanford, CA, United States

**Keywords:** avalanche, climate change, hypoxia, search and rescue, snow, trauma

## Abstract

Avalanches are major natural hazards in snow-covered mountains, threatening people and infrastructure. With ongoing climate change, the frequency and types of snow avalanches may change, affecting the rates of avalanche burial and survival. With a wetter and warmer snow climate, consequences of burial may become more severe. In this review, we assess the potential effects of climate change on the frequency and characteristics of avalanches. We then discuss how these changes might affect the survival rates of subjects buried by avalanches and might influence the responses of search and rescue (SAR) teams and health care providers. While climate change is inevitable, the effects on avalanches remain elusive. The frequency of human triggered avalanches may not change, because this depends largely on the number and behavior of winter recreationists. Blunt trauma and secondary injuries will likely become more frequent as terrain roughness is expected to rise and snow cover to become thinner. Higher snow densities in avalanche debris will likely interfere with the respiration of completely buried victims. Asphyxia and trauma, as causes of avalanche death, may increase. It is unlikely that SAR and health care providers involved in avalanche rescue will have to change their strategies in areas where they are already established. The effects of climate change might foster the expansion of mitigation strategies and the establishment of mountain rescue services in areas subject to increased avalanche hazards caused by changes in snow cover and land use.

## Introduction

**Figure 1:**
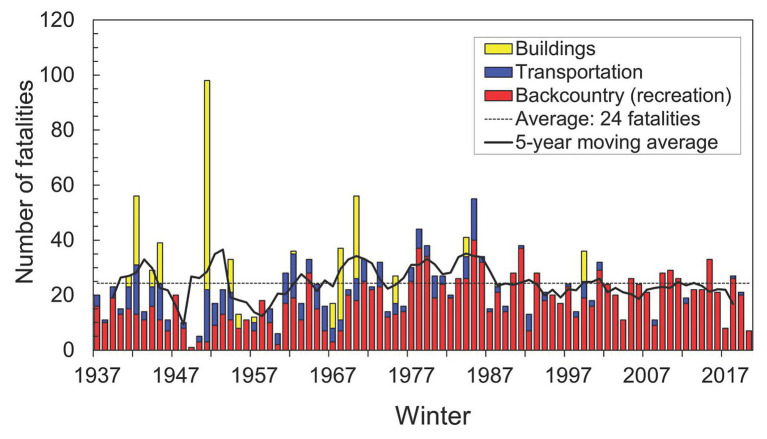
Avalanche fatalities in the Swiss Alps for the period of 1936–1937 to 2019–2020 (84 years). In recent decades, most victims were caught during recreational activities such as skiing (“Backcountry”)—illustrating the emergence and growth of winter tourism. Victims on roads including ski runs are counted in “Transportation.” Victims in villages (“Buildings”) became far less frequent due to extensive mitigation works (updated from Schweizer et al., 2021).

Avalanches are major natural hazards in snow-covered mountain areas, threatening people and infrastructure. Avalanche accidents in the 20th century were characterized by natural disasters and by an increasing number of recreational fatalities following the emergence and growth of winter tourism (Figure 1). Weather events with several days of intense precipitation, both snow and rain, produced numerous catastrophic avalanches in many mountain ranges, affecting inhabited areas and transportation corridors (Brugger et al., 2021). With major investments in mitigation measures, such as defense structures in starting zones and snow sheds to protect infrastructure, especially highways and railways, fatalities in settlements and on roads have decreased in highly populated areas, such as the European Alps (Schweizer et al., 2021). Avalanche accidents affecting populated areas and transport routes are still common in some Asian and South American countries (Brugger et al., 2021). In Europe and North America, avalanche accidents now primarily involve winter recreationists. The increasing popularity of outdoor winter activities has led to increased numbers of accidents (Haegeli et al., 2011; Procter et al., 2014; Techel et al., 2016), most commonly caused by dry-snow slab avalanches (Schweizer and Lütschg, 2001). At least 90% of avalanches that involve injury or death are triggered by recreationists. When victims are completely buried, fewer than half of them survive (Brugger et al., 2001). If not found and extricated within about 35 min, victims die mostly of asphyxia (about 70%), because of obstruction of the upper airway or from the face being surrounded by impermeable snow (Strapazzon and Brugger, 2018). Subjects are more likely to die in high-density avalanche debris (Haegeli et al., 2011; Strapazzon et al., 2017, 2021).

Climate change may affect avalanche accidents and avalanche survival not only in the European Alps and in North America, but also in other mountain regions of the world. With ongoing climate change, the frequency and types of avalanches may change (Hock et al., 2019), affecting the rates of avalanche burial and survival. We first assess how the frequency and types of avalanches might be affected by climate change. We then discuss how these changes might affect the pathophysiology of avalanche burial and the survival rates of subjects buried by avalanches. We discuss how such changes might influence the response of search and rescue (SAR) teams and health care providers. Evidence-based guidelines (Brugger et al., 2013; Truhlar et al., 2015; Van Tilburg et al., 2017) and checklists (Kottmann et al., 2015, 2017) may need to be revised periodically to adapt to changes in the patterns of injuries and death.

## Effects of Climate Change on Mountain Snow Cover

Mountain snow cover is affected by climate change with increasing air temperatures and changes in precipitation. With the general increase of the freezing line in winter, it will more often rain at higher elevations than at present. With this shift from solid to liquid precipitation, seasonal snow lines will be found at higher elevations and snow seasons will be shorter than they are now (Marty et al., 2017; Beniston et al., 2018).

### Temperature

According to the 2019 Intergovernmental Panel on Climate Change (IPPC) special report (Hock et al., 2019), there is medium quality evidence, with a high level of confidence, that temperature increases will cause changes in the frequency, intensity, and types of snowfalls, according to elevation. At lower elevations, the frequency and intensity of snowfall is likely to decrease, causing the snowpack to be thinner and wetter, with a higher average density. The duration and geographic extent of the snowpack will decrease at lower elevations. At moderate and high elevations, changes in temperature and precipitation may be more dynamic, with rapid oscillations between extremes, and with fewer clear trends because of local effects.

For Switzerland, the projected changes to the mountain cryosphere were recently described in detail (CH2018, 2018). Climate projections for the European Alps suggest that the warming trend (+2°C since 1864 for Switzerland) will continue, causing decreased duration, depth, and geographic extent of snow cover, especially below 2,000 m. In Switzerland, since 1970, the number of snow days has already decreased by 50% below 800 m, and by 20% below 2,000 m. Projections for 2060, based on the Swiss climate scenarios (for the unabated emissions scenario RCP8.5; CH2018, 2018), predict a further warming of 2–3.5°C in the winter months with a rise in freezing levels of 400–650 m.

### Precipitation

Precipitation is predicted to increase by 10%, based on the 100-year extreme precipitation event as well as the most intense 1-day precipitation event in a given year. Rain will become more frequent than snow. Precipitation intensity is predicted to increase slightly during major snowstorms. Air temperature during a storm event determines the vertical limit of snowfall. In Switzerland, the main weather pattern responsible for large amounts of snow is strong northwestern flow. Air masses may still be cold enough for intense snowfall, resulting in major avalanche cycles. In Switzerland, snow depth will decrease in general except at elevations above about 2,500 m. The increase at high elevations is related to higher snowfall intensity with increasing air temperature (Frei et al., 2018). Other studies (Kapnick and Delworth, 2013; O’Gorman, 2014; Rajczak and Schär, 2017) have also predicted an increase in winter precipitation extremes, such as intense snowfalls and rain-on-snow events, possibly with an increase in total snowfall.

Overall, the projected changes to the mountain cryosphere suggest a dramatic decrease in snow cover duration and snow water equivalent. However, while the prediction of an increase in air temperature is robust, there remains large uncertainty concerning the effects on winter precipitation (Beniston et al., 2018).

## Effects of Climate Change on Avalanche Activity and Avalanche Dynamics

### Avalanche Activity

Major avalanche cycles in mountains on all continents are related to severe winter storms. Precipitation amounts and air temperatures during storms as well as prior snow stratigraphy affect the frequency and types of avalanches (e.g., Stoffel et al., 1998; Schweizer et al., 2009). While it seems clear how climate change will affect mountain snow cover at lower elevations, changes above treeline (1,800–2,200 m in the European Alps) are less certain. Most avalanche starting zones in the European Alps are located above treeline. The effects on avalanches in response to climate change, therefore remain elusive (Pierce et al., 2008; Kapnick and Hall, 2012; Marty et al., 2017; Najafi et al., 2017; Beniston et al., 2018; Wilbur and Kraus, 2018). A recent assessment emphasizes that future avalanche hazard will depend on interactions between increasing air temperatures and possibly increasing precipitation intensities. Less snow may not necessarily mean fewer avalanches (Reuter et al., 2020). Regional predictions necessary to assess changes in avalanche activity are complicated by model uncertainties, internal climate variability, and complexities of downscaling (Lafaysse et al., 2014; Mankin and Diffenbaugh, 2015).

A common view, expressed with medium confidence in the recent IPPC special report (Hock et al., 2019), is that the number of avalanches and runout distances will decrease at lower elevations. Avalanches involving wet snow, even in winter, will occur more frequently, agreeing with results of a sensitivity study (Martin et al., 2001) that projected slightly decreased avalanche hazard in winter with an increase in the relative proportion of wet-snow avalanches. The first projection on annual and seasonal timescales of future natural avalanche activity predicted a 20–30% reduction in the French Alps from the middle to the end of the 21st century compared to the reference period, 1960–1990 (Castebrunet et al., 2014). The model results indicate that avalanche activity will decrease in spring and at lower elevations, but increase at higher elevations in winter because of more favorable conditions for wet-snow avalanches earlier in the season.

### Avalanche Dynamics

Avalanche flow patterns and consequently runout distances strongly depend on the interactions of snow temperatures with snow cover (Naaim et al., 2013; Steinkogler et al., 2014; Köhler et al., 2018). If very large avalanches that release in dry snow entrain warm snow lower in the avalanche path, the flow characteristics change, producing shorter, less predictable runouts. These trends towards wetter avalanches in runout zones have already been observed and are likely to continue (Eckert et al., 2013; Pielmeier et al., 2013; Naaim et al., 2016). Similar predictions have been made for northern Japan (Katsuyama et al., 2017), and for North America (Lazar and Williams, 2008). In the western Indian Himalaya, a 150-year time series of the occurrence and runout distances of avalanches on a single avalanche slope with several avalanche paths was reconstructed using dendrogeomorphic techniques (Ballesteros-Cánovas et al., 2018). These avalanches threaten a transportation corridor. The tree-ring-based analysis suggested that avalanche occurrences and runout distances have increased in recent decades, which the authors attributed to climate warming. They concluded that their findings contradict the intuitive assumption that warming results in less snow with fewer avalanches (Ballesteros-Cánovas et al., 2018).

The powerful effects of air temperature on avalanche flow regimes were demonstrated in January 2018 and 2019 in Europe. In January 2018, a major storm on the northern slopes of the Alps dropped 2–3 m of snow in <72 h. During the storm, the snowfall limit varied considerably, with rain as high as 2,200 m (Bründl et al., 2019; Bühler et al., 2019). Many very large avalanches that started as dry-snow avalanches lost momentum while continuing through the partially wet snowpack. The flow characteristics transformed to those typical of wet-snow avalanches. Overall, the alterations in flow resulted in shorter runouts. This scenario, with large amounts of new snow at high elevations, rain at lower elevations, and flow alterations was hypothesized to be the new normal that might be typical for decades to come, because of climate change (Stoffel and Corona, 2018). However, only 1 year later, in January 2019 there was another avalanche cycle on the northern slopes of the Alps with colder air temperatures. There were many powder-snow avalanches that ran all the way to the valley bottoms, causing substantially more damage to forests than the avalanche cycle of the previous year. These two avalanche cycles in January 2018 and 2019 demonstrated the way in which temperatures of air masses during major storms can influence the characteristics of avalanches, affecting the level of hazard to people and infrastructure.

### Catastrophic and Human-Triggered Avalanche Hazards

**Figure 2:**
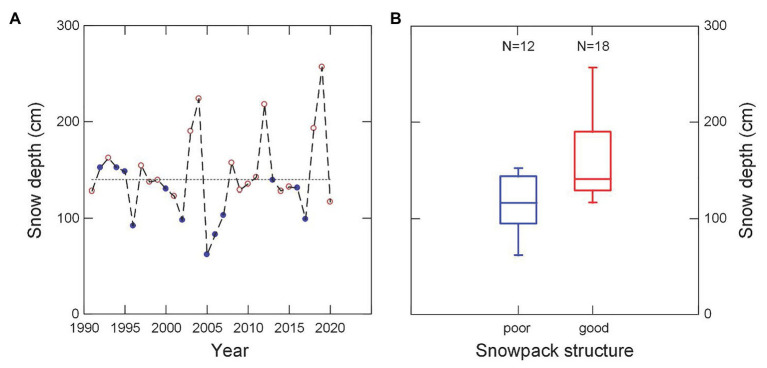
Snowpack structure in relation to snow depth for the Weissfluhjoch study site 2,540 m (Davos, Switzerland) for the 30-year period 1990–1991 to 2019–2020. **(A)** Snow depth in mid-January with full blue circles indicating poor snowpack structure, and red open circles indicating good snowpack structure. Snowpack structure is classified based on snow profile type as described by Schweizer and Wiesinger (2001): “poor” corresponds to profiles types 1–5, (with weak basal layers), “good” to profile types 6–10, (with well-consolidated basal layers). **(B)** Snow depth by snowpack structure class. With poor snowpack structure snow depth is significantly lower than with good structure (median: 117 vs. 141 cm; Mann-Whitney U-Test, *p* = 0.012). Boxes span the interquartile range from 1st to 3rd quartile with a horizontal line showing the median. Whiskers show the range of observed values that fall within 1.5 times the interquartile range above the 3rd and below the 1st quartile.

Catastrophic avalanches generally result from large amounts of cold snow, while avalanches triggered by recreationists are the result of a particular snow stratigraphy: cohesive slab layers above a so-called “weak” snow layer (Gaume et al., 2017). The formation of a weak layer is favored by shallow snow depth and cold, clear weather (Figure 2). These conditions often prevail in early winter, resulting in a snowpack that consists of layers of faceted crystals and depth hoar when large temperature gradients cause recrystallization (e.g., Birkeland et al., 1998). This situation is often observed in winters with scarce snow and more generally in interior alpine regions with continental type snowpacks and will likely become more common with the trend towards generally less snow. The presence of persistent weak layers, represents a major threat for recreationists (Techel et al., 2015; Statham et al., 2018). Avalanche risk caused by natural events will also be influenced by changes in land use, land cover, and demographics (García-Hernández et al., 2017; Giacona et al., 2018) that may be affected by climate change. There will be a higher risk of disastrous events where poorly managed winter tourism activities, transportation routes, and exploitation of natural resources lead to increases in exposure. In contrast, risk will likely decrease in areas of depopulation and abandonment of pastures, with reforestation, adequate land use planning, and measures to mitigate avalanche a hazards.

Trends for future avalanche activity are difficult to predict using climate models. Avalanches are local and extreme events, with highly nonlinear responses to snow and weather conditions (Schweizer et al., 2003). Avalanche dynamics depend on complex relationships between temperatures and snow amounts (Bartelt et al., 2012; Naaim et al., 2013). For the time being, it is unclear whether warmer temperatures will lead to fewer avalanches because of less snow (Beniston et al., 2018) or whether avalanche activity will sometimes be locally more severe because of more intense winter precipitation.

## Effects of Climate Change on Avalanche Accidents

Several decades of reliable observations are required to quantify trends in avalanche activity and avalanche accidents. Avalanche statistics are generally influenced by underreporting of non-fatal accidents and potentially large effects of years with extreme events, single multi-casualty accidents, and random effects. Reliable statistics on avalanche fatalities are available in a few European countries (Austria, France, Germany, Italy, Slovenia, and Switzerland; Techel et al., 2016) and in Canada and the United States in North America. There were 4,750 avalanche fatalities in the European Alps in the 46 years between 1970 and 2015, with no significant trends (Techel et al., 2016). Between 1983 and 2010, the annual number of avalanche fatalities in Canada and the United States was also stable [International Commission for Alpine Rescue (ICAR), 2016]. Winter recreationists account for more than 90% of avalanche fatalities in the European alpine countries (Techel et al., 2016; Höller, 2017) as well as in Canada, and the United States (Haegeli et al., 2011; Jekich et al., 2016).

**Figure 3:**
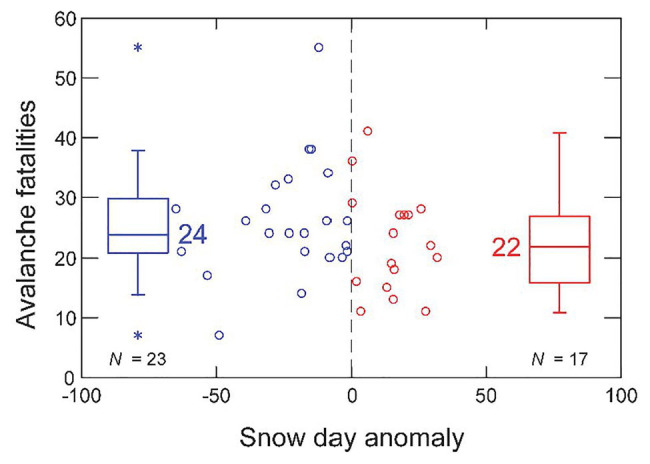
Avalanche fatalities in the Swiss Alps for the 40-year period 1978–1979 to 2017–2018 in relation to snow cover duration, expressed as snow day anomaly. Snow day anomaly refers to the deviation from the mean number of snow days. Snow days are days with snow depth >50 cm for a group of seven measurement stations in the Swiss Alps at 1,300–1,800 m. The 30-year mean (1961–1990) is 89 days (Marty, 2008). Open circles show individual values, in blue for years with low snow and in red for years with high snow. Box plots to left and right show the distribution characteristics. The median numbers of fatalities were 24 in low snow years and 22 in high snow years. The difference between the two groups is not statistically significant (U-Test, *p* = 0.28). Same presentation of box plots as described in Figure 2; asterisks show outliers.

Since the majority of avalanche fatalities now involve winter recreationists, most avalanche accidents occur because of characteristics of the snow cover, such as the presence of weak layers and the presence of recreationists. Even if we expect generally thinner snowpacks, more variable temperatures, and occasional rain-on-snow events to decrease the hazard, the frequency of human-triggered avalanches might not decrease if winter tourism continues to be popular. In general, decreases in the amount of snow may not be associated with fewer avalanche fatalities (Figure 3). The combination of a thin snowpack including persistent weak layers, as a consequence of climate change, and a major storm may also cause a widespread cycle of very large avalanches in which the entire weak snowpack is entrained.

Future developments are uncertain regarding the threats to villages and transportation from avalanches. The risk largely depends on the frequency and intensity of major storm events and future socioeconomic development in mountainous regions.

## Effects of Climate Change on Patterns of Injuries and Avalanche Survival

**Figure 4:**
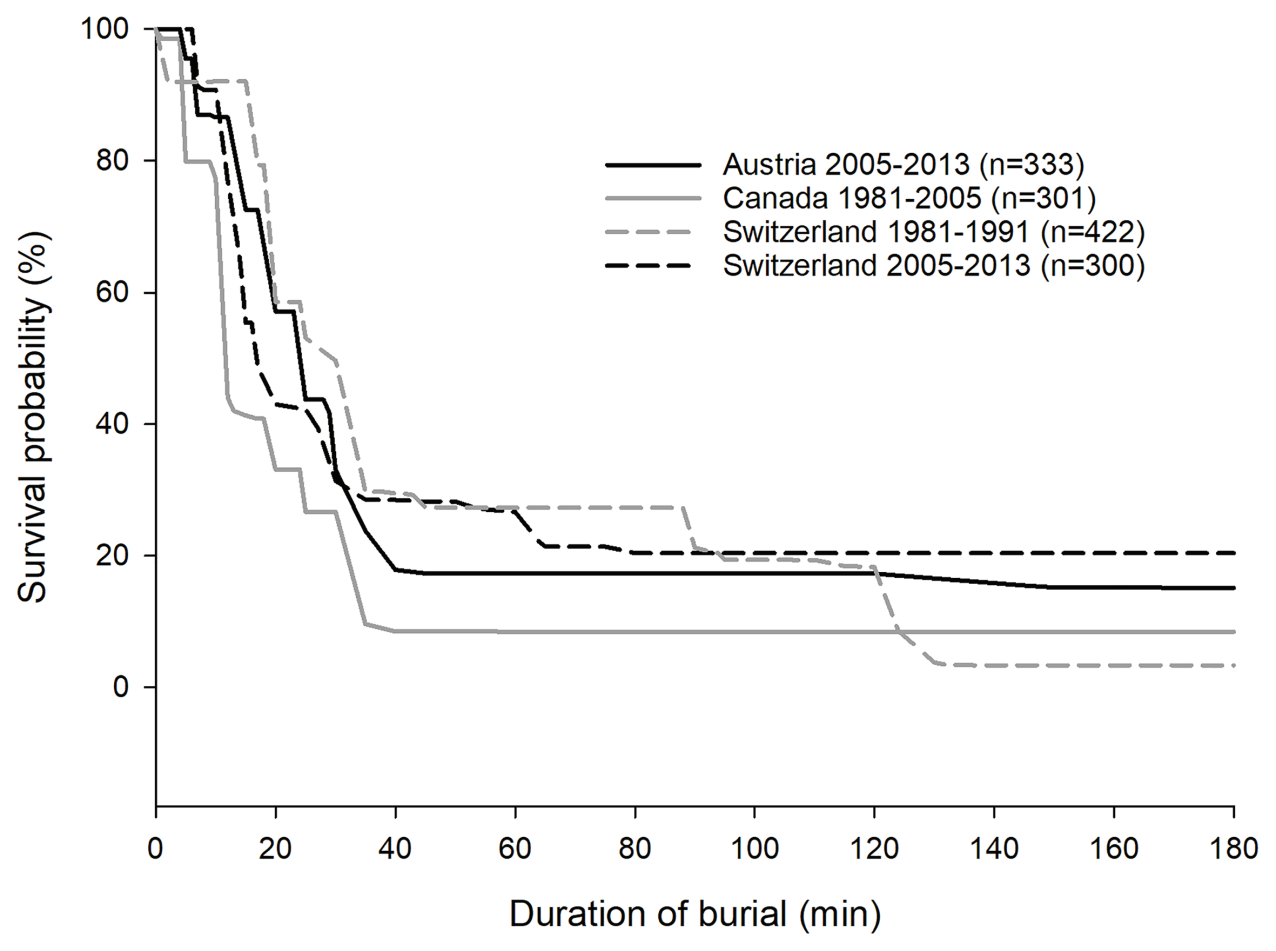
Survival curves for Austria (solid black line: 2005–2013), Canada (solid gray line: 1981–2005), and Switzerland (dashed gray line: 1981–1991; black dashed line: 2005–2013) for completely buried victims (modified from Falk et al., 1994; Haegeli et al. 2011; Procter et al., 2016).

The main determinants of survival in avalanches during winter recreational activities are grade of burial (partial or complete), airway patency in cases of complete burial, and the presence of injuries (Boyd et al., 2010). Survival analysis has shown a mortality rate of about 50% for completely buried avalanche victims and <5% for partially or unburied victims (Brugger et al., 2021). Trauma is the initial cause of death in about 20–30% of victims; asphyxia is the most common cause of death in completely buried victims (about 70%) according to survival curves from Austria, Canada, and Switzerland (Figure 4; Falk et al., 1994; Haegeli et al., 2011; Procter et al., 2016). Variations in survival patterns were related to different snow climates (Haegeli et al., 2011). Climate change may affect the epidemiology of avalanche survival. The characteristics of survival curves result from the combination of climate effects, measures to decrease avalanche accidents, improved medical care, and trends in winter recreational activities. The original avalanche survival curve from Switzerland, using data from 1980 to 1991, was similar to more recent survival curves based on data from 2005 to 2013 (Falk et al., 1994; Brugger et al., 2001; Haegeli et al., 2011; Procter et al., 2016), but there are no studies that investigated whether changes in one factor could mask the effects of other factors.

### Respiration Under Snow

**Figure 5:**
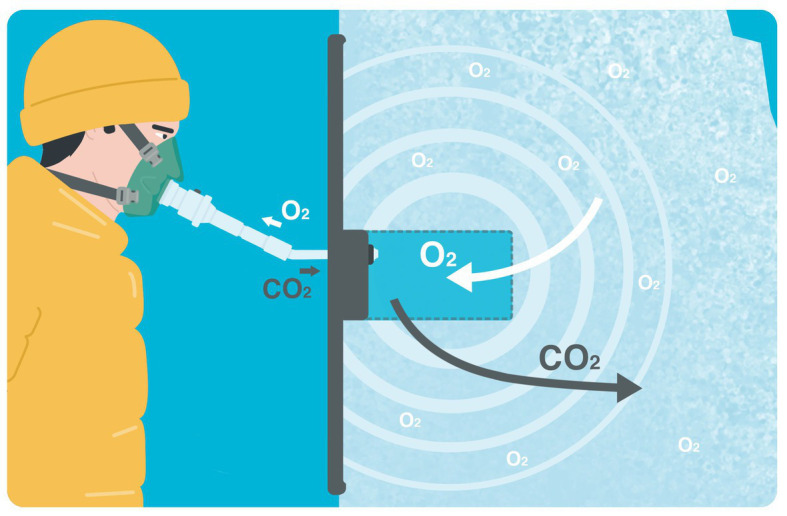
Breathing into an artificial snow avalanche. The porosity of the snow allows diffusion of O_2_ from snow debris into the air pocket and diffusion of exhaled CO_2_ into the surrounding snow. CO_2_, carbon dioxide; O_2_, oxygen (Illustration by Dalila Rovazzani. All rights reserved, used with permission).

The survival curves in continental climates show a later drop compared to those in maritime climates (Haegeli et al., 2011). The snow characteristics and pattern of avalanche debris seem to have direct effects. Asphyxia occurs earlier in snow climates where snow has a higher density. Snow is a highly porous medium containing a large amount of interstitial air. If the upper airway is not obstructed, breathing in medium to low snow density allows longer survival than breathing in high-density snow, as higher oxygen availability slows oxygen desaturation (Figure 5). In a field study, none of participants breathing in an air pocket of 4 L in medium or low-density snow had to stop the trial due to hypoxia (i.e., SpO_2_ ≤ 75%) within 30 min, while about 60% of the trials had to be interrupted when breathing in high snow density (Strapazzon et al., 2017). Survivors of long burials may sustain critically low levels of oxygen and carbon dioxide before hypothermia can protect the central nervous system, especially in snow of low or medium density. Dense snow in avalanche debris is likely to interfere with respiration and oxygenation of completely buried victims (Strapazzon et al., 2017, 2021). In an animal study, pigs without sufficient exchange of respiratory gases became hypoxemic with a mixed respiratory and metabolic acidosis and increased serum potassium (Paal et al., 2013). Wet, higher density snow was associated with rapid depletion of oxygen in human subjects buried in simulated avalanche debris (Strapazzon et al., 2017). Wet snow was also associated with decreased cerebral oxygenation (Strapazzon et al., 2021). These data might partially explain the differing survival curves between maritime and continental climates in Canada (Haegeli et al., 2011). Climate change might lead to wetter snow earlier in winter in continental areas and at higher elevations with lower survival rates in avalanche burials.

Compression by snow seems also to have an effect on survival (Procter et al., 2016). Individuals buried >120 cm are five times more likely to die than those buried ≤40 cm, independent of the duration of burial. As the snowpack is likely to become thinner, this could have an independent positive effect of reducing burial depth and increasing survival. This hypothesis has yet to be investigated.

### Traumatic Injuries

In maritime climates of Canada higher rates of trauma were recorded, associated with wet snow and forested terrain, than in continental climates with dry snow and lower treelines (Haegeli et al., 2011). Blunt trauma from collisions with trees caused 68% of traumatic deaths in a Canadian study (Boyd et al., 2009), but severe injuries can also be caused by impacts with ice or rocks, by churning or crushing effects of snow (Grossman et al., 1989; Boyd et al., 2009; Haegeli et al., 2011). Terrain roughness is expected to rise and snow cover to become thinner, because of climate change. Blunt trauma and secondary injuries will likely become more frequent. Decreased level of consciousness caused by trauma may further decrease survival probability by predisposing to asphyxiation.

### Summer Avalanches

In the Northern hemisphere, summer avalanches occur between June and October. Summer avalanches accounted for 4% of avalanches in Switzerland between 1984 and 2014 (Pasquier et al., 2017). Mortality was higher in summer than in winter avalanches, although there were fewer complete burials and fewer asphyxia-related deaths. Trauma was more common in summer avalanches than in winter avalanches, but hypothermia was less common. There were no trends in fatalities over time, so no evidence-based data currently predict an effect of climate change on summer avalanche events at higher elevations.

### Catastrophic Avalanches

Survival curves of victims buried in buildings or vehicles in catastrophic avalanches are similar to survival curves of winter recreationists (Brugger et al., 2001). The fatality rate of victims involved in catastrophic avalanches has been estimated to be 12%, compared to about 27% for victims involved in avalanches during winter recreational activities (Brugger et al., 2001). Victims of catastrophic avalanches have a lower mortality because they are often protected in vehicles or structures from traumatic injuries. Avalanche victims trapped in buildings or vehicles may have larger air pockets or connections to the outside, resulting in higher survival probabilities than for completely buried victims in unprotected situations. Two women and an 11-year-old child survived for 37 days in a collapsed animal shed after a catastrophic avalanche in 1755 in the Italian Alps (Parry-Jones and Parry-Jones, 1994). If very large avalanches, with wet, dense debris, increase with climate change, mortality in catastrophic avalanches might increase.

## Future Challenges for Search and Rescue Organizations

Search and rescue and health care providers involved in avalanche rescue face logistical and medical challenges. SAR teams often have to mount extensive searches in hazardous terrain. There are multiple victims in about a third of avalanche missions (Mair et al., 2013; Kottmann et al., 2018). In a study from Tyrol, Austria, most avalanche accidents and helicopter SAR operations occurred on days with avalanche danger levels 2 (moderate) or 3 (considerable; Rainer et al., 2008; Mair et al., 2013), i.e., at intermediate hazard when there are more people recreating in the backcountry compared to days with higher danger. Climate change is unlikely to affect these statistics on human-triggered avalanches because the frequency of avalanche accidents depends on the presence and behavior of winter recreationists.

Although in mountain regions, such as Austria, there are always SAR and health care providers on standby, the survival rate in avalanche accidents is about 80% when avalanche victims are extricated by bystanders and <20% when victims are extricated by helicopter SAR teams (Mair et al., 2013). On-scene arrival times, with a mean of about 45 min, are longer than the rapid initial drop in the survival curve at 35 min, after which a completely buried victim with an obstructed airway is no longer able to breathe effectively in avalanche debris. These statistics are unlikely to change significantly with climate change, even if the ratio of wet- to dry-snow avalanches increases. Because most fatal avalanches are triggered by winter recreationists, it will still be critical that people in avalanche terrain be trained in companion rescue, resuscitation, and prevention of avalanche accidents (Procter et al., 2014; Strapazzon et al., 2018; Wallner et al., 2019).

In areas with well-established mountain rescue organizations, such as Europe and North America, an increase in wet-snow avalanches would require more rescue assets, including personnel and equipment, to extricate (or rule out) the presence of buried recreationists. This would cost increased time and money. Extrications from wet-snow and full-depth avalanches require special techniques, more effort, and more resources than extrication from powder avalanches. In some areas, such as coastal and semicoastal areas of Alaska, extremely deep burials are common. Special techniques, such as ground radar, are sometimes used to find bodies and vehicles under snow depths of 20 m or more. Climate change could expand areas where such events could happen, requiring more SAR teams with adequate preparedness.

In areas where avalanches become more frequent, larger, and more destructive, the consequences for local populations might be disastrous, especially in densely inhabited areas. In regions such as the Indian Himalaya, avalanches threaten the population in villages and on roads (Ballesteros-Cánovas et al., 2018). Unlike the European Alps, where most main roads and villages have been protected since the 1960s by structures such as snow fences, barriers, and snowsheds, there are few, if any protective structures in the Himalayas. In many areas, the population is growing and new roads are being built. The impact of catastrophic avalanches on the local populations will likely be greatest in these countries. Unfortunately, there are no organized rescue groups in most areas of the Himalayas, Karakoram, and Andes. In many Asian and South American countries, mountain rescue is performed by the military. In the Nepal Himalayas, most high elevation rescues are performed by climbers already in the area with evacuations by privately-owned helicopter services. Mitigation efforts in these countries should include the construction of protective structures and the establishment of civil protection and mountain rescue services.

## Conclusion

Climate change will significantly affect the duration and extent of seasonal snow cover in mountain regions. While these changes are predictable, the effects on the frequency and characteristics of avalanches remain elusive. The overall frequency of avalanches is likely to decrease. As snow cover decreases at lower elevations, the area where avalanches can occur decreases. At higher elevations, where snowfall is still abundant, and might increase in intensity, changes to the avalanche regime might be less prominent. The frequency of human-triggered avalanches might not change, because this depends mainly on the number of winter recreationists. Blunt trauma and secondary injuries will likely become more frequent as terrain roughness is expected to increase and snow cover to become thinner. As snow density in avalanche debris increases, respiration of completely buried victims will be more limited. Asphyxia and trauma as causes of avalanche death may therefore increase. It is unlikely that SAR and health care providers involved in avalanche rescue will have to change their strategies in areas where they are already established. The effects of climate change might foster the expansion of mitigation strategies and the establishment of mountain rescue services in areas subject to increased avalanche hazards caused by changes in snow cover and land use.

## Author Contributions

GS, JS, IC, MB, HB, and KZ designed the study, performed the literature search, and drafted the manuscript. All authors contributed to manuscript revision, read and approved the submitted version.

### Conflict of Interest

The authors declare that the research was conducted in the absence of any commercial or financial relationships that could be construed as a potential conflict of interest.
